# Brightly Luminescent (Tb_x_Lu_1−x_)_2_bdc_3_·nH_2_O MOFs: Effect of Synthesis Conditions on Structure and Luminescent Properties

**DOI:** 10.3390/molecules28052378

**Published:** 2023-03-04

**Authors:** Viktor G. Nosov, Yulia N. Toikka, Anna S. Petrova, Oleg S. Butorlin, Ilya E. Kolesnikov, Sergey N. Orlov, Mikhail N. Ryazantsev, Stefaniia S. Kolesnik, Nikita A. Bogachev, Mikhail Yu. Skripkin, Andrey S. Mereshchenko

**Affiliations:** 1 Saint-Petersburg State University, 7/9 Universitetskaya emb., 199034 St. Petersburg, Russia; 2 Federal State Unitary Enterprise “Alexandrov Research Institute of Technology”, 72 Koporskoe Shosse, 188540 Sosnovy Bor, Russia; 3 Institute of Nuclear Industry, Peter the Great St. Petersburg Polytechnic University (SPbSU), 29 Polytechnicheskaya Street, 195251 St. Petersburg, Russia; 4 Nanotechnology Research and Education Centre RAS, Saint Petersburg Academic University, ul. Khlopina 8/3, 194021 St. Petersburg, Russia

**Keywords:** metal–organic framework, luminescence, rare earth, terbium, lutetium, antenna effect

## Abstract

Luminescent, heterometallic terbium(III)–lutetium(III) terephthalate metal-organic frameworks (MOFs) were synthesized via direct reaction between aqueous solutions of disodium terephthalate and nitrates of corresponding lanthanides by using two methods: synthesis from diluted and concentrated solutions. For (Tb_x_Lu_1−x_)_2_bdc_3_·nH_2_O MOFs (bdc = 1,4-benzenedicarboxylate) containing more than 30 at. % of Tb^3+^, only one crystalline phase was formed: Ln_2_bdc_3_·4H_2_O. At lower Tb^3+^ concentrations, MOFs crystallized as the mixture of Ln_2_bdc_3_·4H_2_O and Ln_2_bdc_3_·10H_2_O (diluted solutions) or Ln_2_bdc_3_ (concentrated solutions). All synthesized samples that contained Tb^3+^ ions demonstrated bright green luminescence upon excitation into the ^1^ππ* excited state of terephthalate ions. The photoluminescence quantum yields (PLQY) of the compounds corresponding to the Ln_2_bdc_3_ crystalline phase were significantly larger than for Ln_2_bdc_3_·4H_2_O and Ln_2_bdc_3_·10H_2_O phases due to absence of quenching from water molecules possessing high-energy O-H vibrational modes. One of the synthesized materials, namely, (Tb_0.1_Lu_0.9_)_2_bdc_3_·1.4H_2_O, had one of the highest PLQY among Tb-based MOFs, 95%.

## 1. Introduction

Rare earth elements (REE)-based compounds are promising materials for applications in medicine [1,2], sensors [3,4], catalysis [5], anticounterfeiting [6,7], bioimaging [8,9], photovoltaic systems [10,11,12], etc. due to their unique optical and magnetic properties. The positions of the narrow emission bands of REE ions attributed to f–f transitions strongly depend only on the type of lanthanide ions. This property allows photoluminescence color tuning of the REE-containing materials [13]. Usually, purely inorganic compounds of lanthanides demonstrate relatively weak photoluminescence intensity because they possess extremely low extinction coefficients due to the forbidden nature of f–f transitions, which makes the direct excitation of ions inefficient. This issue can be overcome using the so-called “antenna effect”. The antenna effect is realized in some metal–organic compounds in which the light is absorbed by the chromophore group of the organic ligand followed by the energy transfer to the lanthanide ion, which then emits the light corresponding to the characteristic f–f transitions [14,15,16]. The typical ligands used in REE antenna complexes are calixarenes [17], dipicolinic acid [18], tris-bipyridines [19], and carboxylates including terephthalates [20,21,22]. Lanthanide-based metal–organic frameworks (MOFs) combine the optical properties of REE-based materials with the topological features of MOFs, which makes them exceptional materials for chemical sensors [23], optical thermometers [24,25], and OLED components [26,27]. Eu^3+^ and Tb^3+^ ions are often used as activators in such materials because of their strong red and green emissions, respectively [21,28,29,30]. The simultaneous presence of several lanthanide ions in one compound provides the possibility to gain the properties of multimodal imaging agents and allows one to discover the energy transfer mechanisms in such compounds [31,32,33]. Moreover, some studies showed the enhancement of luminescence of Eu^3+^, Tb^3+^, and Sm^3+^ containing antenna MOFs upon dilution with paramagnetic Gd^3+^ ions, whereas the substitution of luminescent REE ions by diamagnetic La^3+^, Y^3+^, and Lu^3+^ ions does not lead to the luminescence intensity increase [20]. It is important to note that the doping by the aforementioned ions does not result in the crystalline phase change in the majority of studies of heterometallic REE MOFs. At the same time, the number of studies of luminescent antenna MOFs containing both luminescent and non-luminescent REE ions is still insignificant in contrast with those of solid solutions of purely inorganic compounds [34,35,36,37,38]. Recently, we studied the optical properties of heterometallic europium–lutetium terephthalates [39]. The luminescence quantum yields of the terephthalate ions were found to be increased with a decrease in the europium concentrations in these compounds. We also observed that the substitution of a large amount of Eu^3+^ for Lu^3+^ resulted in a crystalline phase change from Ln_2_bdc_3_·4H_2_O to Ln_2_bdc_3_ (bdc = 1,4-benzenedicarboxylate). The lifetimes of europium (III)’s ^5^D_0_ excited state were found to be larger by 4–4.8 times in an anhydrous phase with low Eu^3+^ content.

In order to further reveal the doping effect of Lu^3+^ ions on the structural and optical properties of antenna luminescent MOFs, in the current work, we studied bimetallic terbium(III)–lutetium(III) terephthalates as obtained by using two methods.

## 2. Results and Discussion

### 2.1. PXRD Results and Analysis

**Figure 1:**
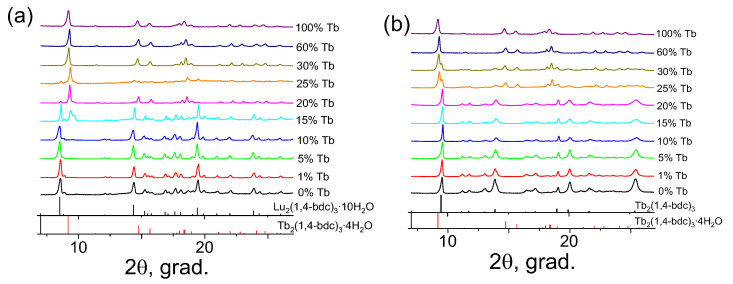
The PXRD patterns of (Tb_x_Lu_1−x_)_2_bdc_3_·nH_2_O (x = 0–1) MOFs synthesized from the diluted (**a**) and concentrated (**b**) solutions as well as the PXRD patterns of Tb_2_bdc_3_·4H_2_O [40], Lu_2_bdc_3_·10H_2_O [41], and Tb_2_bdc_3_ [42] simulated from the single-crystal structures.

All of the syntheses, whatever the chosen method, yielded crystalline samples. In Figure 1a,b, the PXRD patterns of the (Tb_x_Lu_1−x_)_2_bdc_3_·nH_2_O (x = 0–1) MOFs synthesized from diluted and concentrated solutions are shown. We found that all compounds with concentration of terbium (III) ions 30 at. % and more were isostructural to the Ln_2_bdc_3_·4H_2_O crystalline phase (Ln = Ce − Yb) [40], and additional peaks were not observed. At low Tb^3+^ concentrations between 0 and 5 at. %, the positions of the reflexes in the PXRD patterns were different from those of the Ln_2_bdc_3_·4H_2_O and depended on the concentration of the initial reagents (Na_2_bdc, TbCl_3_, and LuCl_3_). Thus, diffraction patterns corresponded to Ln_2_bdc_3_·10H_2_O [41] and Ln_2_bdc_3_ [42] for compounds synthesized from diluted and concentrated solutions, respectively (see Section 3). At intermediate Tb^3+^ concentrations between 10 and 25 at. %, the binary mixtures of the aforementioned crystalline phases were precipitated, namely, Ln_2_bdc_3_·4H_2_O + Ln_2_bdc_3_·10H_2_O and Ln_2_bdc_3_·4H_2_O + Ln_2_bdc_3_ for the (Tb_x_Lu_1−x_)_2_bdc_3_·nH_2_O MOFs obtained from diluted and concentrated solutions, respectively.

**Figure 2:**
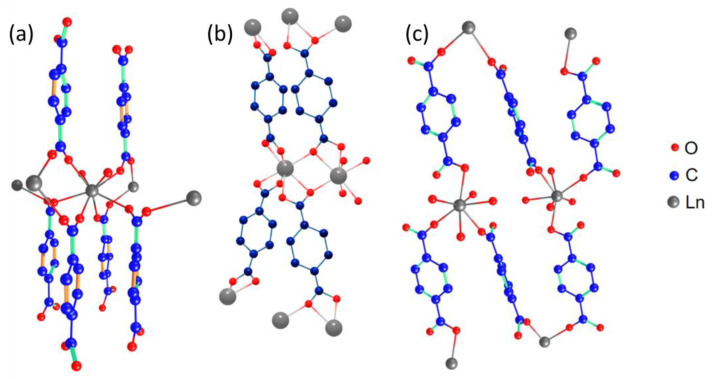
The crystal structures of Tb_2_bdc_3_·4H_2_O (**a**), Tb_2_bdc_3_ (**b**), and Lu_2_bdc_3_·10H_2_O (**c**) generated from the single-crystal diffraction data [40,41,42].

The effect of molar ratio of reagents taken for synthesis on MOFs structure was observed previously (see, for example, [43,44]), but was not explained properly. It is generally accepted that MOFs are formed stepwise from the secondary building units (SBUs), metal–ligand oligomers that replicates themselves to form MOF-like structures [45]. Therefore, the final structure of coordination polymer allows us to assume the possible reasons behind the differences between compounds of two synthesized series. The crystal structures of Ln_2_bdc_3_·4H_2_O, Ln_2_bdc_3_, and Ln_2_bdc_3_·10H_2_O are shown in Figure 2. In Ln_2_bdc_3_·4H_2_O lanthanide (III), ions were bound to two water molecules and six terephthalate ions through oxygen atoms, where Ln^3+^ coordination number (CN) is equal to 8. In Ln_2_bdc_3_ structures, Ln^3+^ ions with CN = 7 coordinated solely to oxygens of terephthalate ions. In the Ln_2_bdc_3_·10H_2_O structure, the metal center coordination number was also equal to 7, but four coordination sites were occupied by water molecules. Two water molecules per one formula unit in the Ln_2_bdc_3_·10H_2_O structure were located in interplanar channels. Commonly, Tb^3+^ ions have relatively larger coordination numbers than Lu^3+^ ions. For example, in aqueous solutions, Tb^3+^ dominantly exists in a nonacoordinated form as the [Tb(H_2_O)_9_]^3+^ complex [46], but smaller Yb^3+^ and Lu^3+^ ions possess lower coordination numbers and exist as [Ln(H_2_O)_8_]^3+^ [47,48]. Therefore, we expected that terbium ions will reveal larger coordination numbers than lutetium ion in our MOFs. Indeed, the analysis of the aforementioned structures (Figure 2) revealed that lanthanide ions have coordination numbers of seven in Ln_2_bdc_3_ and Ln_2_bdc_3_·10H_2_O, which are formed in pure lutetium terephthalate, and in (Tb_x_Lu_1−x_)_2_bdc_3_·nH_2_O at high Lu^3+^ content levels. In Ln_2_bdc_3_·4H_2_O, which is formed in pure terbium terephthalate and in mixed Tb–Lu terephthalates at high Tb^3+^ content levels, the coordination number of Ln^3+^ ions is equal to eight. The reasons that can explain the difference between structures of lutetium terephthalates synthesized from diluted (Lu_2_bdc_3_·10H_2_O) and concentrated (Lu_2_bdc_3_) solutions are unclear. We assume the key factor that affects the structure of precipitated MOF is the fractional distribution of initially formed metastable complexes [Ln(H_2_O)_x_(bdc)_y_]^3−2y^ [49]. These complexes then aggregate into SBUs, which further form MOFs. Apparently, in concentrated solutions, complexes have higher Lu^3+^:bdc^2−^ ratios (1:2 or 1:3) than in diluted solution (1:1). Therefore, further formed SBUs and MOFs of Lu_2_bdc_3_ had larger number of coordinated oxygens of terephthalate ligands than Lu_2_bdc_3_·10H_2_O.

In our previous work, we reported the similar behavior of the (Eu_x_Lu_1−x_)_2_bdc_3_·nH_2_O MOFs obtained from concentrated solutions [39]. We found that phase transition occurred at significantly lower Eu^3+^ concentrations (6 at. % of Eu^3+^ vs. 30 at. % of Tb^3+^). This observation can be explained by the lower ionic radius of Tb^3+^ (1.040Å) than that of Eu^3+^ (1.066Å) [50]. The structure with CN = 7 (Ln_2_bdc_3_) is more advantageous for Tb^3+^ than for Eu^3+^, which forms a structure Ln_2_bdc_3_·4H_2_O with larger CN = 8 beginning at 6 at. % of Eu^3+^ ions.

### 2.2. Thermogravimetric Analysis (TGA)

**Figure 3:**
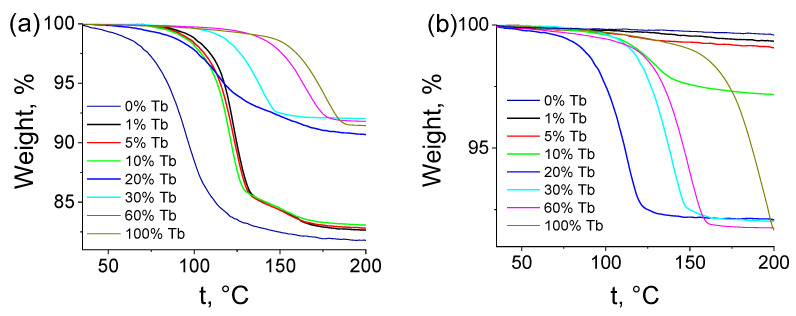
Thermogravimetric analysis (TGA) curves showing the weight loss profile of (Tb_x_Lu_1−x_)_2_bdc_3_·nH_2_O materials synthesized from the diluted (**a**) and concentrated (**b**) solutions during thermal decomposition.

The thermal behavior of the selected compounds (Tb_x_Lu_1−x_)_2_bdc_3_·nH_2_O (x = 0–1) was studied by using the thermogravimetric method (TGA). The TGA curves of the MOFs obtained from diluted and concentrated solutions were recorded in the temperature range of 35–200 °C (Figure 3). When heated, the lanthanide terephthalates decomposed in two common steps: (i) dehydration of the compounds, resulting in formation of Ln_2_bdc_3_ at about 100–200 °C, and (ii) the structural decomposition of coordination polymers [42]. The observed weight loss at 100–190 °C for all measured samples corresponded to the dehydration step; therefore, the analysis of the TGA curves allowed us to calculate the average numbers of water molecules in the coordination polymers (Tb_x_Lu_1−x_)_2_bdc_3_·nH_2_O.

**Figure 4:**
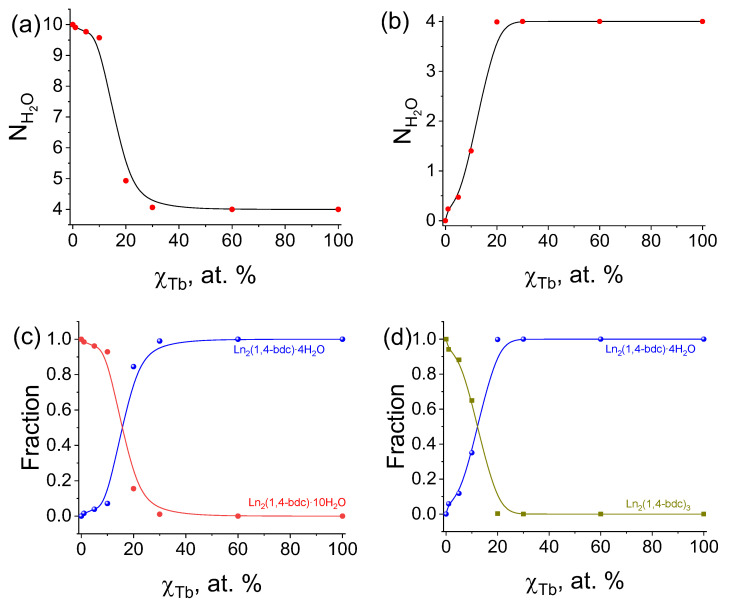
The number of water molecules per one formula unit calculated from TGA data for (Tb_x_Lu_1−x_)_2_bdc_3_·nH_2_O materials synthesized from the diluted (**a**) and concentrated (**b**) solutions; the molar fractions of Ln_2_bdc_3_·4H_2_O and Ln_2_bdc_3_·10H_2_O (**c**) and the molar fractions of Ln_2_bdc_3_·4H_2_O and Ln_2_bdc_3_ (**d**) as functions of Tb^3+^ concentration for (Tb_x_Lu_1−x_)_2_bdc_3_·nH_2_O materials synthesized from the diluted and concentrated solutions, respectively.

The number of water molecules per one formula unit N(H_2_O) for all selected compounds as function of Tb^3+^ concentration is shown in Figure 4a,b for samples synthesized from diluted and concentrated solutions, respectively. The number of water molecules per one formula unit is equal to four for pure terbium terephthalate (100 at. % Tb^3+^) in both series. The N(H_2_O) value increases from 4 to 10 upon the substitution of Tb^3+^ by Lu^3+^ ions (decreasing of Tb^3+^ content) in (Tb_x_Lu_1−x_)_2_bdc_3_·nH_2_O MOFs obtained from the diluted solutions (Figure 4a). However, for MOFs synthesized from the diluted solutions, the number of water molecules decreases from four to zero upon Tb^3+^ concentration decrease (Figure 4b). These facts are in agreement with the XRD data, in which we observed phase transitions from Ln_2_bdc_3_·4H_2_O either to Ln_2_bdc_3_·10H_2_O or Ln_2_bdc_3_ upon the decrease of Tb^3+^ content. Summarizing the TGA and XRD data, we estimated the molar fraction of each coexisting crystalline phase (Figure 4c,d). The molar fraction of Ln_2_bdc_3_·4H_2_O increased from 0 to 30 at. % Tb^3+^ for two synthesized series of MOFs (Tb_x_Lu_1−x_)_2_bdc_3_·nH_2_O, and simultaneously, the molar fraction of the second coexisting phase decreased. In the Tb^3+^ concentration range of 30–100 at. %, only Ln_2_bdc_3_·4H_2_O was present in both series.

### 2.3. Luminescent Properties

Aromatic carboxylate ions, especially benzene dicarboxylates, are typical linkers for the luminescent antenna MOF design [15,20] due to the efficient sensitization of lanthanide luminescence. The sensitization mechanism consists of several steps. Upon UV-photon absorption, the linker is promoted into the S_n_(^1^ππ*) exited electronic state, which is followed by the fast internal conversion to S_1_(^1^ππ*). Due to the heavy atom effect, the S_1_ state of the linker efficiently undergoes intersystem crossing to the T_1_(^3^ππ*) triplet electronic excited state [32]. If the T_1_ state of organic linker lies slightly higher in energy than one of the levels of activator lanthanide ion, then the energy is efficiently transferred to the lanthanide ion and followed by the photon emission corresponding to the f–f transition. Thus, terbium terephthalate, Tb_2_bdc_3_·4H_2_O, demonstrates a relatively high Tb^3+^ photoluminescence quantum yield (43–55% [32,42,51]) upon UV-excitation into terephthalate ions due to the fact that the T_1_ state of the terephthalate ion (E(T_1_) ≈ 20,400–20,650 cm^−1^ [32] for bdc^2-^) lies only 50—300 cm^−1^ above the ^5^D_4_ level of the Tb^3+^ ion (E(^5^D_4_) ≈ 20,350 cm^−1^ [52]).

**Figure 5:**
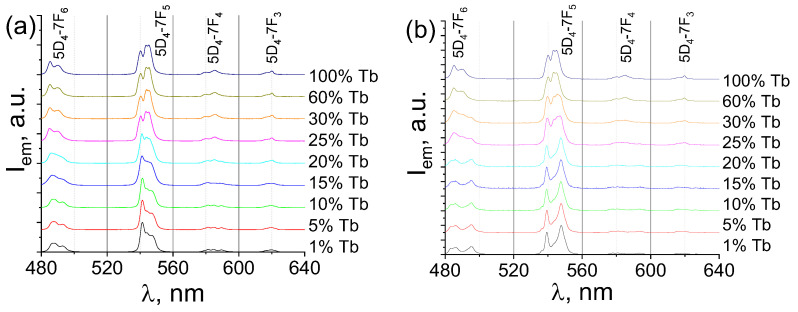
Emission spectra of (Tb_x_Lu_1−x_)_2_bdc_3_·nH_2_O materials synthesized from the diluted (**a**) and concentrated (**b**) solutions upon 280-nm excitation at the selected Tb^3+^ concentrations.

The emission spectra of the synthesized compounds, which were measured upon 280-nm excitation into the S_n_(^1^ππ*) excited electronic state of terephthalate ions, are shown in Figure 5. The observed emission spectra are typical for compounds containing Tb^3+^ ions [53] and consist of narrow bands corresponding to ^5^D_4_→^7^F_J_ (J = 3–6) transitions of Tb^3+^: ^5^D_4_→^7^F_6_ (≈491 nm), ^5^D_4_→^7^F_5_ (≈543 nm), ^5^D_4_→^7^F_4_ (≈585 nm), and ^5^D_4_→^7^F_3_ (≈620 nm). One can observe that the fine structure of Tb^3+^ emission spectra of (Tb_x_Lu_1−x_)_2_bdc_3_·nH_2_O significantly changes at Tb^3+^ concentration of about 20 at. % in both studied series. It is well-known, that the fine structure of lanthanide (III) ions strictly depends on the local symmetry of emitting lanthanide ions [54,55,56,57]. Indeed, one can notice three different types of fine structure of the spectra: (i) compounds with terbium (III) content of 25 at. % and more in both series (corresponding to the (Tb_x_Lu_1−x_)_2_bdc_3_·4H_2_O structure that dominates in this range of concentrations); (ii) MOFs with Tb^3+^ concentrations less than 25 at. % in series obtained from diluted solutions ((Tb_x_Lu_1−x_)_2_bdc_3_·10H_2_O as the dominating structure); (iii) compounds with terbium (III) concentrations less than 25 at. % in series obtained from concentrated solutions ((Tb_x_Lu_1−x_)_2_bdc_3_ as the dominating structure). The difference is that the fine structure of the emission bands is attributed to the different symmetry of the first coordination sphere of the Tb^3+^ ion in these three types of crystalline structures.

**Figure 6:**
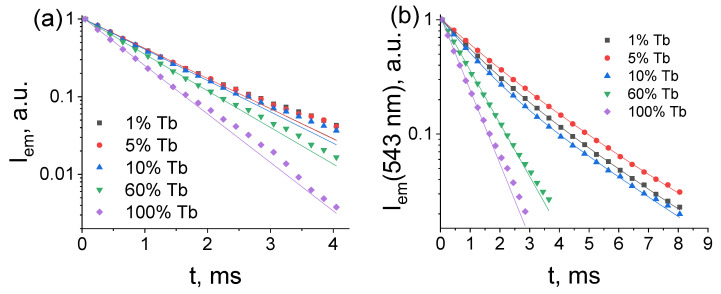
The photoluminescence decay curves of (Tb_x_Lu_1−x_)_2_bdc_3_·nH_2_O materials synthesized from the diluted (**a**) and concentrated (**b**) solutions upon UV-excitation at the selected Tb^3+^ concentrations.

Figure 6 displays the photoluminescence decay curves measured upon UV-excitation of (Tb_x_Lu_1−x_)_2_bdc_3_·nH_2_O MOFs synthesized via the two methods mentioned as monitored at 543 nm (^5^D_4_→^7^F_5_ transition). At terbium (III) ion concentrations of 60 and 100 at. %, photoluminescence decay curves were well-fitted with the single exponential functions (Equation (1)) with time constants τ of about 0.7–1.1 ms. At low levels of Tb^3+^ content (1, 5, and 10 at. %), the photoluminescence decay curves of the compounds obtained from concentrated solutions fit the double exponential functions (Equation (2)). The biexponential behavior of the photoluminescence decay indicates the presence of different relaxation pathways of Tb^3+^ ions corresponding to two terbium ions with different coordination environments. We believe that the larger time constant τ_2_, which is about 2.6–3 ms (Table 1), corresponds to lifetime of ^5^D_4_ state Tb^3+^ ions in the (Tb_x_Lu_1−x_)_2_bdc_3_ structure. The smaller time constant τ_1_ value (1.0–1.5 ms) can be assigned to the lifetime of the ^5^D_4_ state of terbium (III) ions in the (Tb_x_Lu_1−x_)_2_bdc_3_·4H_2_O structure. The photoluminescence decay curves of the (Tb_x_Lu_1−x_)_2_bdc_3_·nH_2_O compounds with x = 0.01, 0.05, and 0.10, which were obtained from diluted solutions, fit the single exponential functions (eq. 1) with time constants of about 1.1 ms. As the XRD and TGA data shows the coexistence of Ln_2_bdc_3_·4H_2_O and Ln_2_bdc_3_·10H_2_O phases in these compounds, one would expect the presence of two different exponential components of photoluminescence decay curves. Most likely, the values of the ^5^D_4_ energy level lifetime of Tb^3+^ ions in Ln_2_bdc_3_·4H_2_O and Ln_2_bdc_3_·10H_2_O structures are close to each other, as pseudo-single-exponential decay was observed.

(1) $$I=I_{1}\cdot e^{-\frac{t}{\tau }}$$

(2) $$I=I_{1}\cdot e^{-\frac{t}{\tau_{1}}}+I_{2}\cdot e^{-\frac{t}{\tau_{2}}}$$

We have found that the ^5^D_4_ excited state lifetimes in the (Tb_x_Lu_1−x_)_2_bdc_3_·nH_2_O MOFs obtained from diluted solutions decreased from 1.122 to 0.696 ms with the increase of terbium concentration due to the increased probability of energy transfer between neighboring Tb^3+^ ions with subsequent quenching of impurities. At the same time, the photoluminescent quantum yields (PLQY) of these compounds had maxima at about 60 at. % of Tb^3+^, where PLQY is equal to 60% (Table 1). Typically, emission intensity and PLQY nonlinearly depend on the concentration of Tb^3+^ ions [58,59]. This type of concentration dependence can be explained by the two competitive effects in REE-containing phosphors [60,61]. Thus, the rise of the numbers of luminescent sites results in radiative emission probability increased and, as a result, the emission intensity and PLQY increased. At the same time, upon the Tb^3+^ concentration’s rise, the distance between Tb^3+^ ions decreased, resulting in the nonradiative processes probability increase that led to the emission quenching [62], resulting in lower PLQY values of pure terbium terephthalate (100 at.% of Tb^3+^) relative to the MOFs containing 60 at.% of Tb^3+^. The PLQY of the (Tb_x_Lu_1−x_)_2_bdc_3_·nH_2_O MOFs obtained from concentrated solutions are equal to the ones obtained from the diluted solutions at the Tb^3+^ concentration of 60 and 100 at. %, where the MOFs formed in the same crystalline phase, namely, Ln_2_bdc_3_·4H_2_O. A further decrease of Tb^3+^ content in the MOFs obtained from the diluted solutions resulted in a significant PLQY rise, reaching maxima of 95% for the (Tb_0.1_Lu_0.9_)_2_bdc_3_·1.4H_2_O sample. The higher values of PLQY and excited state lifetimes of these materials are attributed to the formation of the anhydrous Ln_2_bdc_3_ crystalline phase. The PLQY of the Ln_2_bdc_3_ MOFs were significantly higher than that of the Ln_2_bdc_3_·4H_2_O and Ln_2_bdc_3_·10H_2_O MOFs due to the absence of water molecules coordinated to Tb^3+^ ions, which efficiently quenches luminescence due to energy transfer from the ^5^D_4_ excited state of Tb^3+^ ions to the high-energy O-H stretching vibrational modes of H_2_O molecules [63].

## 3. Materials and Methods

Benzene-1,4-dicarboxylic (terephthalic, H_2_bdc) acid (>98%), sodium hydroxide (>99%), nickel(II) chloride hexahydrate (>99%), EDTA disodium salt (0.05M aqueous solution), and murexide were purchased from Sigma-Aldrich Chemie GmbH (Taufkirchen, Germany) and used without additional purification. Lutetium (III) nitrate pentahydrate and terbium (III) nitrate pentahydrate were purchased from Chemcraft (Kaliningrad, Russia). The 0.3 M solution of disodium terephthalate (Na_2_bdc) was prepared by dissolving 0.6 moles of sodium hydroxide and 0.3 moles of terephthalic acid in 1 L of distilled water. Volumes of 0.2 M of TbCl_3_ and LuCl_3_ solutions were prepared and standardized using back complexometric titration. Thus, 1 mL of LnCl_3_ (Ln = Tb, Lu) solution with a concentration of about 0.3 M, 20 mL of 0.05 M EDTA, 10 mL of ammonium buffer solution (pH = 9), and a pinch of murexide indicator were taken in a conical flask. The obtained solution was titrated with 0.05 M NiCl_2_ [64]. Then, standardized LnCl_3_ solutions were diluted to 0.2 M.

White powders of the (Tb_x_Lu_1−x_)_2_bdc_3_·nH_2_O MOFs were synthesized by the direct mixing of two aqueous solutions: (1) sodium terephthalate and (2) terbium and lutetium nitrates taken in various ratios, as shown in Table 2. In order to reveal the effect of the concentrations of the initial solutions on the properties of the obtained materials, we synthesized two series of (Tb_x_Lu_1−x_)_2_bdc_3_·nH_2_O MOFs. Series 1 was obtained from the Na_2_bdc and LnCl_3_ diluted solutions, where 8 mL of 0.1 M Na_2_bdc solution was added dropwise under vigorous stirring to a solution containing 5 mL of distilled water and 2 mL of 0.2M TbCl_3_ and LuCl_3_ solutions taken in certain ratios (Table 2). Series 2 was obtained from the Na_2_bdc and LnCl_3_ concentrated solutions, where 3 mL 0.3 M Na_2_bdc solution was rapidly added to the 2 mL of TbCl_3_ and LuCl_3_ solutions taken in various ratios, as shown in Table 2. Obtained suspensions were kept for one 1 h at room temperature, and then, solid precipitates of the (Tb_x_Lu_1−x_)_2_bdc_3_·nH_2_O MOFs were separated from the reaction mixture via centrifugation (2300 g) and washed with deionized water 5 times. The resulting white powders of terbium-lutetium terephthalates were dried in an air atmosphere at 60 °C for 24 h.

The Tb^3+^/Lu^3+^ ratios in the synthesized (Tb_x_Lu_1−x_)_2_bdc_3_·nH_2_O compounds were confirmed with energy-dispersive X-ray spectroscopy (EDX) (EDX spectrometer EDX-800P, Shimadzu, Japan) (Table 3). We found that the amounts of the elements are consistent with experimental EDX data. The X-ray powder diffraction (XRD) data of obtained (Tb_x_Lu_1−x_)_2_bdc_3_·nH_2_O samples were taken with a D2 Phaser (Bruker, Billerica, MA, USA) X-ray diffractometer using Cu K_α_ radiation (λ = 1.54056 Å). The thermal behavior of the compounds was studied via thermogravimetry using a Thermo-microbalance TG 209 F1 Libra (Netzsch, Selb, Germany) with a heat-up rate of 10 °C/min. To carry out photoluminescence studies, the synthesized samples (20 mg) and potassium bromide (300 mg) were pressed into pellets (diameter 13 mm). Solid-state luminescence emission spectra were recorded with a Fluoromax-4 fluorescence spectrometer (Horiba Jobin–Yvon, Kyoto, Japan). Lifetime measurements were performed with the same spectrometer using a pulsed Xe lamp (pulse duration 3 µs). The quantum yield measurements were performed by using the Fluorolog 3 Quanta-phi device (Horiba Jobin–Yvon, Kyoto, Japan).

## 4. Conclusions

In this work, we reported the phase composition and the optical properties of luminescent antenna MOFs: heterometallic terbium(III)–lutetium(III) terephthalates. The series of (Tb_x_Lu_1−x_)_2_bdc_3_·nH_2_O (x = 0–1) were synthesized via direct reaction between aqueous solutions of disodium terephthalate and nitrates of corresponding lanthanides with two methods: using diluted and concentrated solutions. At Tb^3+^ concentrations more than 25 at. %, synthesized compounds existed in the Ln_2_bdc_3_·4H_2_O crystal structure with the coordination number (CN) of the lanthanide ion equal to eight. Lu^3+^ ions typically have lower coordination numbers than Tb^3+^ ions; hence, at high lutetium (III) content, structures with CN(Ln^3+^) < 8 crystallized. Therefore, compounds containing small amounts of terbium (III) ions formed in crystalline phases different from Ln_2_bdc_3_·4H_2_O. (Tb_x_Lu_1−x_)_2_bdc_3_·nH_2_O (x = 0–0.01) compounds, synthesized from concentrated solutions, dominantly existed in the Ln_2_bdc_3_ crystal structure with CN(Ln^3+^) = 7. (Tb_x_Lu_1−x_)_2_bdc_3_·nH_2_O (x = 0–0.01) MOFs obtained from diluted solutions formed as Ln_2_bdc_3_·10H_2_O crystalline phases with CN(Ln^3+^) = 7. At 2–25 at. %, Tb^3+^ ion binary mixtures of the aforementioned crystalline phases were observed. All of the synthesized samples containing Tb^3+^ ions demonstrated admirable green luminescence upon 280nm excitation due to the ^5^D_4_→^7^F_J_ (J = 3–6) transitions of the Tb^3+^ ions. Upon UV-photon absorption, terephthalate ion was promoted into the S_n_(^1^ππ*) excited electronic state, which was followed by the fast internal conversion to S_1_(^1^ππ*) and then to the T_1_(^3^ππ*) triplet electronic excited state via efficient intersystem crossing due to the presence of the heavy lanthanide ion. The T_1_ state of the terephthalate ion lies slightly higher in energy than the ^5^D_4_ level of the Tb^3+^ ion, resulting in the efficient energy transfer to this level that was followed by radiative ^5^D_4_→^7^F_J_ (J = 3–6) transitions. The Tb^3+^ ions in Ln_2_bdc_3_·4H_2_O, Ln_2_bdc_3_·10H_2_O, and Ln_2_bdc_3_·10H_2_O crystal structures demonstrated different fine structures in their emission bands due to the different local symmetry of the Tb^3+^ ions in these three types of crystalline structures. The ^5^D_4_ excited state lifetimes and photoluminescence quantum yields of (Tb_x_Lu_1−x_)_2_bdc_3_ (x = 0.01, 0.5, 0.1) compounds were significantly larger than for samples of (Tb_x_Lu_1−x_)_2_bdc_3_·4H_2_O (x = 0.6, 1) and (Tb_x_Lu_1−x_)_2_bdc_3_·10H_2_O (x = 0.01, 0.5, 0.1) due to the absence of the luminescence quenching of the Tb^3+^ by coordinated water molecules. Meanwhile, we cannot rule out effect of the crystalline structure on the relative energies of the T_1_(^3^ππ*) triplet’s electronic excited state and the ^5^D_4_ level of Tb^3+^ ions, which affect the efficiency of the T_1_-to-^5^D_4_ energy transfer efficiency, resulting in PLQY changes. As a result of our study, we synthesized the material, namely (Tb_0.1_Lu_0.9_)_2_bdc_3_·1.4H_2_O, which has one of the highest PLQY among Tb-based MOFs, 95%.

## Figures and Tables

**Table 1:** Lifetimes (τ) and photoluminescence quantum yields (Φ_PL_) of (Tb_x_Lu_1−x_)_2_bdc_3_·nH_2_O materials at the selected Tb^3+^ concentrations synthesized from the diluted (Series 1) and concentrated (Series 2) solutions.

Series 1 (From Diluted Solutions)			Series 2 (From Concentrated Solutions)			
Χ_Tb_ (at. %)	τ, ms	Φ_PL_, %	Χ_Tb_ (at. %)	τ_1_, ms	τ_2_, ms	Φ_PL_, %
1	1.12 ± 0.02	38	1	1.17 ± 0.04	2.63 ± 0.10	77
5	1.12 ± 0.02	56	5	1.53 ± 0.05	3.00 ± 0.24	88
10	1.08 ± 0.01	58	10	1.02 ± 0.03	2.61 ± 0.08	95
60	0.92 ± 0.02	60	60	0.94 ± 0.02		60
100	0.70 ± 0.01	49	100	0.69 ± 0.01		49

**Table 2:** The volumes of the initial TbCl_3_ and LuCl_3_ solutions used for the synthesis of (Tb_x_Lu_1−x_)_2_bdc_3_·nH_2_O MOFs.

Χ_Tb_ (at. %)	V(0.2M TbCl_3_), mL	V(0.2M LuCl_3_), mL
0	0.00	2.00
1	0.02	1.98
5	0.10	1.90
10	0.20	1.80
15	0.30	1.70
20	0.40	1.60
25	0.50	1.50
30	0.60	1.40
60	1.20	0.80
100	2.00	0.00

**Table 3:** Tb^3+^ atomic fractions (relative to the total amount of Tb^3+^ and Lu^3+^) in (Tb_x_Lu_1−x_)_2_bdc_3_·nH_2_O compounds synthesized from the diluted (Series 1) and concentrated (Series 2) solutions. Measurements were taken during synthesis and obtained from EDX data.

Series 1 (from Diluted Solutions)		Series 2 (from Concentrated Solutions)	
Χ_tb_ (At. %), Taken	Χ_Tb_ (%), EDX	Χ_Tb_ (at. %), Taken	Χ_Tb_ (%), EDX
0	0	0	0
1	0.74 ± 0.07	1	0.70 ± 0.07
5	4.6 ± 0.5	5	4.6 ± 0.5
10	9 ± 1	10	10 ± 1
15	15 ± 3	15	14 ± 1
20	19 ± 2	20	20 ± 2
25	26 ± 3	25	23 ± 2
30	29 ± 3	30	27 ± 3
60	57 ± 5	60	57 ± 5
100	100	100	100

## Data Availability

The data presented in this study are available in the article.

## Sample Availability

Samples of the compounds are available from the authors.
